# Autophagy: A New Avenue and Biochemical Mechanisms to Mitigate the Climate Change

**DOI:** 10.1155/2024/9908323

**Published:** 2024-10-12

**Authors:** Muhammad Abubakkar Azmat, Malaika Zaheer, Muhammad Shaban, Saman Arshad, Muhammad Hasan, Alyan Ashraf, Muhammad Naeem, Aftab Ahmad, Nayla Munawar

**Affiliations:** ^1^Department of Plant Breeding and Genetics, University of Agriculture Faisalabad, Sub-Campus Burewala 61010, Vehari, Pakistan; ^2^Department of Agricultural Biotechnology, Ondokuz Mayis University, Samsun 55270, Turkey; ^3^Texas A&M University, College Station, TX, USA; ^4^Pakistan Environmental Protection Agency (Pak-EPA), Ministry of Climate Change and Environmental Coordination, Islamabad, Pakistan; ^5^College of Life Science, Hebei Normal University, Shijiazhuang 050024, China; ^6^Biochemistry/Center for Advanced Studies in Agriculture and Food Security (CAS-AFS), University of Agriculture, Faisalabad, Pakistan; ^7^Department of Chemistry, College of Science, United Arab Emirates University, Al-Ain 15551, UAE

**Keywords:** abiotic stress, ATGs (autophagy-related genes), autophagosome, autophagy, biotic stress, selective autophagy

## Abstract

Autophagy is a preserved process in eukaryotes that allows large material degeneration and nutrient recovery via vacuoles or lysosomes in cytoplasm. Autophagy starts from the moment of induction during the formation of a phagophore. Degradation may occur in the autophagosomes even without fusion with lysosome or vacuole, particularly in microautophagosomes. This process is arbitrated by the conserved machinery of basic autophagy-related genes (ATGs). In selective autophagy, specific materials are recruited by autophagosomes via receptors. Selective autophagy targets a vast variety of cellular components for degradation, i.e., old or damaged organelles, aggregates, and inactive or misfolded proteins. In optimal conditions, autophagy in plants ensures cellular homeostasis, proper plant growth, and fitness. Moreover, autophagy is essential during stress responses in plants and aids in survival of plants. Several biotic and abiotic stresses, i.e., pathogen infection, nutrient deficiency, plant senescence, heat stress, drought, osmotic stress, and hypoxia induce autophagy in plants. Cell death is not a stress, which induces autophagy but in contrast, sometimes it is a consequence of autophagy. In this way, autophagy plays a vital role in plant survival during harsh environmental conditions by maintaining nutrient concentration through elimination of useless cellular components. This review discussed the recent advances regarding regulatory functions of autophagy under normal and stressful conditions in plants and suggests future prospects in mitigating climate change. Autophagy in plants offers a viable way to increase plant resilience to climate change by increasing stress tolerance and nutrient usage efficiency.

## 1. Introduction

Plants are continuously subjected to various recurrent but transient stresses; therefore, they have evolved complex systems to balance the cellular homeostasis during development and environmental changes to complete their life cycle in the best possible way [1]. Autophagy is an intracellular catabolic pathway that degrades cytoplasmic material, such as old or damaged organelles, undesirable proteins, and infectious agents in large quantities. It delivers cargoes of autophagy to lysosomes and vacuoles in mammals and yeast/plants respectively for breakdown and recycling [2, 3]. The response of autophagy is normally slow during plant growth and development to maintain homeostasis, but it is increased to address various environmental challenges such as starvation, senescence, extreme temperatures, dehydration, salinity, phytohormone-activated cellular reprogramming, immunological response, and pathogen infection [4, 5]. Autophagy is categorized into different types based on the mechanism involved, the specific cargo targeted for degradation, and the cellular context in which it occurs. Macroautophagy, microautophagy, organelle-specific autophagy, chaperone-mediated autophagy, and mega-autophagy are some major autophagy types [6]. Microautophagy is triggered when the tonoplast invades the core vacuole directly and vacuolar hydrolases destroy an isolated body containing cytoplasmic components [7]. In mammals, CMA (chaperone-mediated autophagy) is not induced through the autophagosome formation, rather Chaperone protein Hsc70 is used to identify cytoplasmic substrates, which are then sent to the lysosome for breakdown [8]. In plant development and pathogen attack, mega-autophagy solely aids in PCD (programmed cell death), during which the tonoplast is destroyed and hydrolases are secreted in the cytoplasm. Mega-autophagy is an extreme form of massive degradation, resulting in cell death [9, 10].

Macroautophagy requires the synthesis of cytoplasmic autophagosomes (double-membrane vesicles) from the precursor phagophore. Autophagy-related genes control the production of a membrane structure (just like cup shaped) at the phagophore assembly site [11]. Several cellular organelles are involved in the production of phagophore, i.e., mitochondria, endoplasmic reticulum (ER), ER-mitochondria contact sites, and plasma membrane [4, 12]. The phagophore surrounds cytosolic molecules to produce autophagosomes [13]. Although autophagy was previously assumed to be a non-selective breakdown of cellular structures, emerging research revealed that autophagy works as a component of stress responses of plants and helps in cellular quality regulation. It is quite selective in targeting old or damaged components [14]. Several ATG genes have been linked to both bulk and selective autophagy, implying that they may share a common autophagy mechanism [15].

In this review, we investigated the recent advancement in understanding the molecular mechanism underlying autophagy in plants and along with its substantial roles in promoting plant growth and responding to stress responses.

## 2. Mechanism of Autophagy in Plants

The molecular mechanism of autophagy was initially observed in yeast. Later, it was explored in other organisms including mammals and plants. In 1950s, autophagosomes were detected for the first time in the mammalian cells [2, 5]. In *Arabidopsis thaliana* (At), the model plant, around forty ATG genes (*AtATGs*) have been found, the majority of which are comparable to yeast ATGs [16–18].

**Figure 1:**
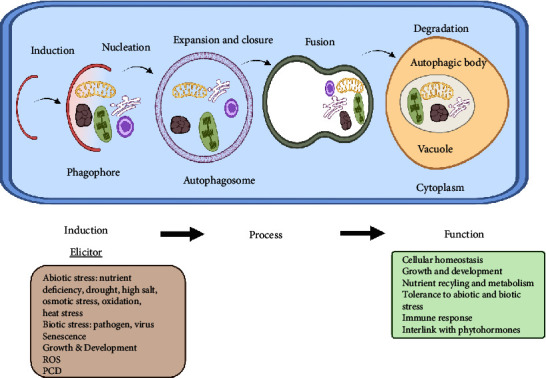
Autophagy induction, process, and functions in plants. In the presence of elicitors, the formation of phagophore is initiated (induction) in plant cell. After processing, autophagosome is formed (process) and then autophagic bodies are degraded (function).

ATG proteins are divided into four functional groups which are explained as follows:

a. ATG1/ATG13 kinase complex: in response to nutrient shortage, it triggers the formation of autophagosome.
b. Specific class III phosphatidylinositol (PI) 3-kinase
c. ATG9 complex: it promotes the expansion of phagophore.
d. ATG8/ATG12: it is a conjugation system just like ubiquitin. During the phagophore expansion and maturation, these systems play their roles [19, 20]. The steps involved during autophagy are highlighted in Figure 1.

## 3. Autophagy: Induction, Vesicle Nucleation, and Autophagosome Formation

Environmental stresses activate autophagy in plants through the ATG1/ATG13 complex and integration of signals from TORs (targets of rapamycin) kinase signaling cascade [2, 21]. TOR is an upstream regulator and a type of serine/threonine kinase that regulates autophagy and belongs to the phosphatidylinositol kinase family [4, 22]. Normally, *Arabidopsis* TOR becomes active and keeps its substrate, ATG13, in a hyper phosphorylated state, which prevents ATG13 from binding to ATG1 [23]. ATG13 is dephosphorylated when nutrients are scarce, which causes TOR to become inactive and causes ATG13 and ATG1 to interact. The complex that mediates the induction of autophagy is created when ATG1-ATG13 connects with the other two subunits ATG11 and ATG101. The combination of ATG1-ATG13-ATG11-ATG101 activates the nucleation of vesicle, phagophore development during autophagy [18, 24]. The VPS34 catalytic subunit of P13K complex (yeast's autophagic class III phosphatidylinositol 3-kinase) controls the induction of vesicles. VPS34 causes both ATG6 and VPS15 to be produced. In *Arabidopsis*, one VPS34 and VPS15 homolog is thought to be present and necessary for kinase function [1, 25]. ATG14 is an autophagic component limited by the PI3K complex that has yet to be found in *Arabidopsis*. In yeast, the amount of autophagy is predicted by ATG6/Beclin1. ATG6 serves as a platform for PI3K activity and is necessary for the autophagosomal vesicles. Homologs of ATG6, PI3K, VPS15, and UVRAG have been found in plants, but these complexes are poorly understood [26].

The complex of ATG9-ATG2-ATG18 complex is involved in the autophagosomes formation and expansion of phagophore by transporting extra lipids to the phagophore [27, 28]. Through an ubiquitin-like conjugation, the expanding phagophore membrane is concurrently filled with ATG8 phosphatidylethanolamine (PE) [29]. Similar to the ubiquitin-activating enzyme (E1), the cysteine protease ATG4 transforms immature ATG8 precursors to matured ATG8 [30]. ATG7 (ATP dependent) activates the matured ATG8. A combination of the ubiquitin-ligase ATG12, ATG5, and ATG16 enzymes (E3) subsequently attaches PE after the ubiquitin-conjugating enzyme (E2)-like ATG3 has transferred ATG8 [26, 31]. ATG12 forms a complex with ATG5 and ATG16 in the ATG5-12-16 order. ATG7 creates ATG8-ATG7 and ATG12-ATG7 intermediates, which move to ATG3 and ATG10, respectively, and then to PE and ATG5, after the activation of the two ATGs. In *Arabidopsis*, a single gene produces the enzyme ATG10, which is similar to E2 [32, 33]. ATG8 is known as an autophagosome marker and is crucial for the precise identification of autophagy payloads. Finally, the vacuolar membrane and the autophagosome's outer membrane fuse, releasing the payloads for oxidation in the lumen of vacuole [13]. The protein complexes, proteins, and their functions in yeast to *Arabidopsis* are shown in Table 1.

## 4. Selective Autophagy in Plants

**Figure 2:**
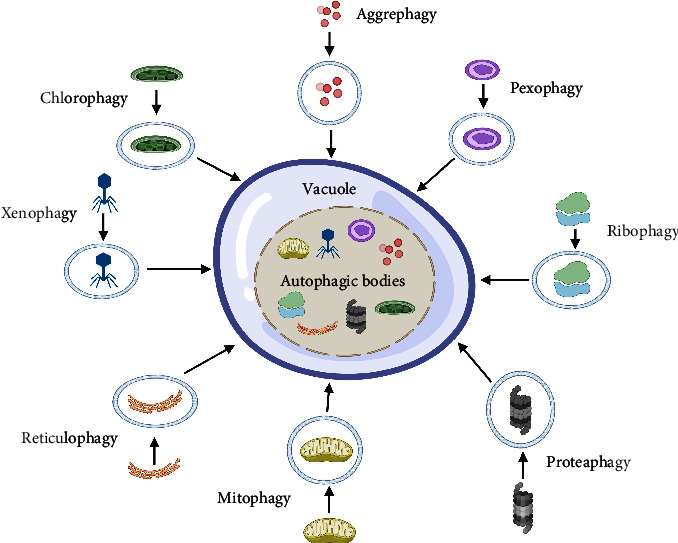
Pictorial representation of different types of selective autophagy such as chlorophagy, mitophagy, ribophagy, reticulophagy, proteaphagy, aggrephagy, and xenophagy in plants.

Plants engage in autophagy at all phases of growth and development. However, the importance of selective autophagy is recently recognized [15, 34]. Chlorophagy, mitophagy, pexophagy (yeasts), aggrephagy, ribophagy, and reticulophagy are the mechanisms by which plants target specific cellular components and organelles. Plants can also use xenophagy to fight viruses. Proteaphagy is a new type of autophagy in which proteasomes are destroyed in response to nitrogen deprivation and proteasome inhibition [9, 35]. In cellular homeostasis, protein and organelle quality regulation, selective autophagy plays a vital role. It enables the cargoes to be recruited into autophagosomes [36, 37]. There are many kinds of autophagy in plants, as shown in Figure 2.

Chloroplasts are photosynthesis-producing organelles found in green plants and algal cells. Chlorophagy is a selective breakdown of chloroplasts by autophagy, and it is a critical quality check for nutrient recycling [38]. In oxidized leaves, chloroplasts are transferred to vacuoles for recycling during hunger and senescence via an autophagy mechanism [20, 39]. In *atg5* and *atg7* mutants, stress-induced chlorophagy is defective, resulting in enhanced ROS (reactive oxygen species) buildup and cell death induced by UVB, hence chlorophagy acts as a photoprotector [6, 40]. Plant peroxisomes are ubiquitin-organelles involved in the B-oxidation of fatty acid, photorespiration, glyoxylate cycle, and other metabolic activities [4]. In early plant growth, these peroxisomes are necessary for the germination of seed, and also involved in synthesis of ROS during oxidative stress [41]. Specific receptors related to pexophagy have been found in various species such as *Pichia pastoris* (ATG30), *Homo sapiens* (NBR1 and p62), and *Saccharomyces cerevisiae* (ATG36) [36].

The autophagic degradation of mitochondria in plants is termed as mitophagy [42]. *Arabidopsis atg11* mutant plants showed same symptoms to well-characterized *atg* mutants during dark-induced leaf senescence, as well as reduced mitochondrial vacuole transport during autophagy [24, 33]. In the lumen of endoplasmic reticulum (ER), unfolded proteins got developed; those phenomena are termed as ER stress or reticulophagy, as a result of unfolded protein responses (UPRs) failing to respond to protein homeostasis within the ER during environmental stresses [43]. Numerous ER-membrane-related receptors that mediate ER tubule and sheet recycling orthologs are present in plants, indicating that these proteins are involved in plant ER recycling [44]. When *Saccharomyces cerevisiae* is starved of nitrogen, it undergoes selective autophagy, which results in the breakdown of ribosomes, called ribophagy [36, 45]. Autophagy-dependent rRNA turnover pathways have been revealed in *Arabidopsis*, but ribophagy in plants has yet to be discovered [46].

Proteaphagy, a mechanism in which latent 26S proteasome complexes are eliminated, was discovered in *Arabidopsis* [47]. The most evident link between these two processes is ubiquitylation. For protein cleavage, it is a universal signal by proteasome and autophagy [48]. In the presence of an inhibited proteasome, which becomes extensively ubiquitylated, the ubiquitin receptors RPN10 mediate proteaphagy. Three ubiquitin-connecting motifs (UIMs) are present in the C-terminal region of RPN10 [49]. Aggrephagy is a kind of selective autophagy in which non-functional proteins are degraded within aggregates [50]. Ubiquitylated insoluble proteins accumulate when NBR1 is mutated in *Arabidopsis* under heat stress. NBR1 and ATG8 could make linkage with cytoplasmic protein aggregates in response to heat stress. Hence, NBR1 is a crucial aggrephagy receptor for preserving proteostasis [37, 51]. Xenophagy refers to the utilization of selective autophagy as a viral defense. Autophagy during plant virus infection is currently limited as compared to animals although the process is comparable [52, 53]. In *Arabidopsis*, NBR1 increases xenophagic destruction of cauliflower mosaic virus particles, reducing aggregation of virus and restricting TuMV [54, 55]. These forms of selective autophagy make sure that a plant's cell can effectively get rid of specific damaged or useless cellular particles in order to maintain its viability and allow plants to survive in challenging environments. The various types of selective autophagy, cargo, adapters, and their functions in environmental stresses are elaborated in Table 2.

## 5. Autophagy Regulation in Plants

**Figure 3:**
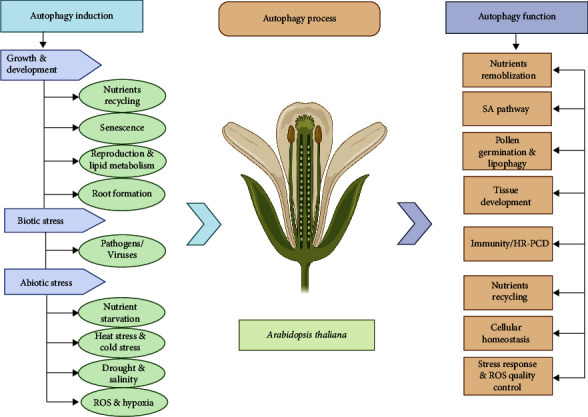
Regulation of autophagy in plants. Autophagy induces in plants from germination to adult and involves in growth and development. It plays important role to mitigate climate change during biotic and abiotic stresses. After processing, autophagy performs many functions such as nutrient remobilization, SA pathway, pollen gemination and lipophagy, tissue development, immunity, nutreints recycling, cellular homeostasis, stress response, and ROS quality control.

In plants, several developmental processes depend on a basic amount of autophagy during normal growth conditions, particularly during specific morphological transitions. In addition, certain environmental conditions trigger autophagy to regulate plant development [20, 58]. Autophagy is important in coping with different sort of biotic and abiotic stresses [4, 59]. In plants, various infections from different pathogens, dietary deficiencies, salinity, drought stress, hypoxia, heat, cold stress, and selective organelle destruction in autophagy are shown in Figure 3.

## 6. Role of Autophagy in the Regulation of Plant Growth and Development

Autophagy has been extensively studied in relation to stress responses in plants although it is essential for keeping homeostasis in a regular state [60]. Autophagy-related genes have been extensively studied for their roles in growth, development, senescence, and nutrient deprivation response in *Arabidopsis*. Most of the mutants of *Arabidopsis*, i.e., *atg4*, *atg5*, *atg7*, *atg10*, and *atg12* exhibit hypersensitivity to nitrogen or carbon shortage and early senescence in comparison to wild type (WT). These *atg5*, *atg7*, and *atg10* seedlings exhibit significantly slower leaf development and growth in addition to chlorotic leaves when nitrogen is depleted. Similarly under carbon starvation *atg4*, *atg5*, *atg7*, and *atg12* exhibit similar abnormalities [31]. However, overexpressing *AtATG5*, *AtATG7*, and *AtATG8* enhances grain filling and nitrogen remobilization efficiency via promoting autophagic activity in *Arabidopsis*, showing useful roles of autophagy in plant productivity [29, 61].

Numerous ATG genes were activated in older leaves of *Arabidopsis*, suggesting that autophagy involve in senescence. Senescence is initiated in *Arabidopsis* leaves by the transcription of *AtATG2*, *AtATG5*, *AtATG7*, *AtATG8*, *AtATG9*, and *AtATG18a* [62]. In *Arabidopsis atg* mutants, several defense-related genes, such as pathogenesis-related gene1 (*AtPR1*), *AtPR2*, and *AtPR5*, and various senescence-associated genes are constitutively expressed [63]. However, salicylic acid (SA) biosynthesis can prevent these mutants early leaf senescence, indicating that the SA pathway is necessary for senescence mediated by autophagy [63, 64]. Beyond *Arabidopsis*, substantial amount of *AtATG8* lipidation was observed within the area of fading senescent maize leaves as a sign for increased autophagy activity. *Arabidopsis* senescence-induced mitophagy is significantly influenced by *AtATG11* [24].

Autophagy plays an essential role in plant reproductive development. For example, postmeiotic tapetum cells have autophagosome structures and lipid bodies that are vacuole-enclosed during pollen formation. The autophagy-defective mutant *Osatg7* lacks these structures. In addition, the mutant exhibits full male sterility and unable to collect materials (lipid and starch) in the pollen grains, demonstrating autophagy's role in tapetum cells and its crucial importance during rice development [65]. The endogenous concentrations of the cytokinin trans-zeatin and gibberellins (GAs) in the *Osatg7* mutant's anthers were much less than those in their WT, suggesting that autophagy might be involved in metabolism of phytohormones during the process of anther development of rice [65, 66]. ATGs were reported to increase in siliques during seed development in *Arabidopsis*. Furthermore, seed embryos have been found to have GFP-ATG8-labeled autophagosome [67]. Similarly, during seed development, autophagy genes *AtATG6/VPS30, AtVPS15*, and *AtVPS34* could regulate nutrient supply [59, 68]. Autophagy is necessary for the zinc and manganese transportation to the seeds in *Arabidopsis* [69]. The germination of tobacco (*Nicotiana tabacum*) pollen requires autophagy, according to a new study [70]. Autophagy is also involved in lipid metabolism called lipophagy but less characterized in plants than yeast and mammals. For example, lipid droplets (LDs) in the tapetum carrying triacylglycerols (TAGs) are essential during pollen maturation. It has been discovered that rice tapetum cells contain LDs that are encased in vacuoles, and *Osatg7* and *Osatg9* mutants' cytoplasm contain more LD-like structures than WT cells, suggesting that plants may also digest LDs [65].

It is crystal clear from the current studies that autophagy involves in the development of plant roots. Under phosphate deprivation, auxin/indole-3-acetic acid (AUX/IAA) proteins are ubiquitylated by phosphorylation and activation of PUB9 (E3 ligase) via ARK2. Following their selective targeting to the autophagosome for degradation, these auxin repressors then release auxin response factors (ARFs) to encourage auxin synthesis and lateral growth of root [71]. Recently, it was discovered that autophagy promotes the development of root meristem which is regulated by glucose. In glucose rich environments, reduction in root meristem occurs due to the oxidation of active IAA via production of ROS. While, in the *atg* mutants, peroxisomes are not able to receive the high-glucose signal, which reduces the buildup of ROS-oxidized indole acetic acid and promotes the development of root [72]. Autophagosome assembles in the WT root seedlings of *Arabidopsis*. The roots of *Arabidopsis thaliana* hydrotropic curvature were discovered to be significantly influenced by autophagy, after moving to normal medium-water stress medium (NM-WSM). This system exhibits hydrotropic curvature in the absence of the *atg2*, *atg5*, *atg8b*, *atg8i*, and *atg9* mutants. H_2_O_2_ builds up in the root curvature at the same rate as autophagosomes during the hydrotropic reaction, suggesting that oxidative stress regulates autophagy during the hydrotropic response in NM-WSM [73]. In the primary and secondary stages of root or stem development in *Populus trichocarpa*, the accumulation of autophagosome during xylem differentiation in roots was observed [74]. In short, autophagy performs distinct physiological processes in plants at various developmental stages as shown in Table 3.

## 7. Autophagy for Defense Against Biotic Stresses

Pathogens or viruses frequently attack plants, which can activate inherent defenses of the host. The pathogen can be identified as the source of the signal via cytoplasmic or membrane-localized receptors, causing an immunological reaction [110]. Pathogens are divided into the following two groups according to their lifestyles: biotrophs and necrotrophs. Biotrophs get their nourishment from the plant host's living tissues and necrotrophs rely on dead host tissue. In contrast, hemibiotrophs are biotrophic as well as necrotrophic at different infection stages [26]. *Botrytis cinerea* is a necrotrophic fungus that infects *Arabidopsis* and causes autophagy. Autophagy-deficient mutants are more susceptible to *Botrytis cinerea* and *Alternaria brassicicola* than WT plants. WRKY33 is a transcriptional factor required for necrotrophic pathogen resistance that interacts with the autophagy protein *AtATG18a*. Plant defense responses to necrotrophic diseases are regulated in a synergistic manner by WRKY33 and jasmonate-mediated signaling pathways [80].

In biotrophic pathogens, autophagy plays a more intricate role than in necrotrophic pathogens. Autophagy is activated in both infected and uninfected parts of the tobacco (*Nicotiana benthamiana*) plants when it is infected with the tobacco mosaic virus (TMV). In addition, autophagy suppressed the growth of pathogens by encouraging hypersensitive response-induced programmed cell death (HR-PCD). BECILIN1 regulates HR-PCD in plants. For example, tobacco ATG6/BECLIN1 is required for PCD restriction in TMV-infected areas [78–80]. In *N. benthamiana*, cell death spread and leaf chlorosis were exacerbated when the autophagy mechanism was compromised by inactivating *NbATG6/NbBeclin1*, *NbPI3K/NbVPS34*, *NbATG3*, or *NbATG7* [76]. Besides HR diseases, plants with impaired autophagy showed premature senescence and cell death, demonstrating a new kind of PCD autophagy-dependent HR in response to AvrRpm1 effector carrying Pst DC3000 that is mainly independent of the *AtMC1*-mediated PCD pathway [35, 78, 82]. The *atg5* knockout mutants and *Arabidopsis atg6* RNAi mutant plants both produced the same results [82]. Similar to this, when avirulent bacterial strain Pst DC3000 infected the *atg7* and *atg9* mutants, necrosis was decreased and resistance was increased (*AvrRps4*). *Arabidopsis* mutant plants showed greater resistance to the virulent bacterial strain *Pst DC3000* or avirulent *Hyaloperonospora arabidopsis* as compared to WT plants, presumably due to a higher salicylic acid (SA) levels in the *atg* mutants, suggesting that autophagy functions in pathogen infection depend on the SA pathway [47, 83]. The HR suppressor NPR1 is a SAR regulator whose protein levels are SA-regulated and tightly controlled by the proteasome via SA receptors NPR3 and NPR4. ATG8-PE and ATG12-ATG5 are thought to control autophagosome formation by affecting NPR3 and NPR4 [55]. Basic resistance of plants to necrotrophic and biotrophic diseases is variably impacted by autophagy. In comparison to the WT, *AtATG5*, *AtATG10*, and *AtATG18a* knockdown or knockout mutants exhibit increased necrosis, increased ROS buildup, and decreased expression of PLANT DEFENSIN 1.2s (*AtPDF1.2s*) are all indicators of enhanced sensitivity to necrotrophic fungal pathogen infection [77].

Plants must adjust autophagy to deliver the proper degree of defense, and some diseases affect that equilibrium to encourage infection. Bax Inhibitor 1 (NbBI1) links with *NbATG6* in *N. tabacum* to control autophagosome biogenesis [81]. In *Arabidopsis*, autophagy regulates both pro- and antipathogen roles during *CaMV* and *Pst DC3000* infection [54, 85]. Recent research indicates that autophagy interacts with pathogen components directly and communicates with defensive signaling pathways like SA and jasmonate to contribute to plant immunity [84, 87]. In *N. benthamiana*, the virulence factor *β*C1 of *Cotton leaf curl Multan virus (CLCuMuV)* interacts with *NbATG8f* to cause plant autophagy and undergo autophagic destruction, hence lowering virulence [84]. Because of plant autophagy's antiviral properties, several viruses have developed defense against autophagy and could promote infection. Barley Stripe Mosaic Virus, for instance, causes *NbATG7-NbATG8* interactions are eliminated by *γ*b factor binding *NbATG7* competitively to prevent autophagy and resulting in harsh consequences [86]. These findings depict that how autophagy plays an important role in the interactions between plants, viruses, and pathogens.

## 8. Role of Autophagy Under Abiotic Stresses

Plants need to adapt under diverse environmental changes, such as lack of nutrients, salt stress, drought stress, heat stress, and oxidative stress for sustainable growth and development [92]. Autophagy is increased in cellular recycling and nutrition remobilization in response to hunger. RNAi silencing of *AtATG18a* and mutant plants of *atg5* and *atg9* under nitrogen deprivation were found to have considerably lower ^15^N remobilization than WT plants [64]. The *atg12* mutant in corn grows more slowly and produces fewer seeds when nitrogen is deficient. Compared to WT, *atg12* had considerably decreased seed output and ^15^N reallocation into the seeds, even in nitrogen-rich environments [89]. In rice, *Osatg7-1* mutant respond in nitrogen remobilization by reduced leaf growth and photosynthetic capability [20]. Recent research has demonstrated that overexpressing *ATG18a* in apples (*Malus pumila*) increases their tolerance to nitrogen deficiency via increases autophagy activity [90]. Furthermore, due to a carbon scarcity, the *Arabidopsis atg5*, *atg7*, *atg6*, *atg9*, and *atg4* mutants show delayed growth, lower protein levels, and increased respiration, suggesting that autophagy is crucial in cellular metabolism and homeostasis [88, 93]. A recent research reveals that a direct phosphorylation of *AtATG6* by *AtSnRK1* during chronic carbon depletion, but not when there is a short-term carbon or nitrogen shortage, initiates an *AtATG1*-independent autophagy route [91].

In plant cells, autophagy is also engaged in the recycling of micronutrients like sulphur and zinc [94–96]. Zinc restriction causes the development of auto-phagosomes in *Arabidopsis*. Genetic testing reveals that *AtATG5* and *AtATG10* accelerate senescence in the *Arabidopsis* during zinc deficiency [94]. More recently, two separate investigations revealed that autophagy is essential for sulphur metabolism. It has been discovered that the *Arabidopsis atg5* mutants have impaired sulphur remobilization from rosettes to seeds [95]. Moreover, the selective autophagy receptor *AtNBR1* is presumably essential for the responses of plants to sulphur deficiency [96].

One of the greatest threats to plant development is heat stress, causes normal proteins to be misfolded and denaturized, which can then be destroyed through autophagy [111]. The accumulation of insoluble and oxidized proteins occurs as a result of heat or cold stresses, triggers autophagy [99]. The heat-stressed proteins are degraded via selective autophagy controlled by NBR1. It has been revealed that *AtATG8* precipitates in plants during heat stress with various types of heat shock proteins (HSP90s, HSP101, and short HSP17.6). These HSPs accumulate when autophagy is dysfunctional [112]. It is evident that several mutants of the genes *atg2-1*, *atg5-1*, *atg7-2*, and *atg10-1* severely affect pollen formation in *Arabidopsis*, indicating that autophagy plays a role in pollen production under high-temperature stress [113]. Tomato plants were less tolerant of heat stress and express fewer ATGs when WRKY33 expression was down regulated, stating that WRKY33 acts in heat stress response via controlling autophagy through transcription [105, 114].

Cold stress disrupts membrane integrity and causes ROS to accumulate, which triggers autophagy [97]. The removal of ubiquitinated proteins by NEXT TO BRA1 GENE1-mediated selective autophagy is additionally required for tomato chilling resistance. The primary signaling factor *BRASSINAZOLERESISTANT 1* (*SlBZR1*) is stabilized in response to the chilling stress in tomato, enhancing NBR1-mediated selective autophagy [59, 98]. Furthermore, cold stress enhances ATG transcript levels and auto-phagosome formation in peppers, but multiple *ATGs* were blocked in tobacco [99].

Environmental challenges that plants frequently experience include salt and drought stresses. The overexpression of *AtATG18a* during drought circumstances served as the first evidence in *Arabidopsis* that autophagy regulates drought stress [3, 115]. In response to salt and drought stress, *AtATG8* in *Arabidopsis* and *OsATG10b* in rice are both engaged [100, 106]. In addition, *atg5*, *atg7*, and *ATG18a* RNAi mutants in *Arabidopsis* exhibit hypersensitization to drought stress [105]. In contrast, overexpressing *MpATG18a* in apples increases both drought tolerance and autophagy activity, highlighting the crucial role of autophagy in drought responses [90, 108]. Interestingly, NADPH oxidase inhibitors can prevent autophagy from being induced by food deprivation and salt stress in contrast to osmotic stress, implying mechanisms that are NADPH oxidase-dependent or -independent can trigger autophagy and also implicated in the oxidative stress response [115]. The *Arabidopsis* gene called *AtCOST1* (CONSTITUTIVELY STRESSED 1) negatively influences drought resistance via regulating autophagy in plant growth. *Arabidopsis cost1* mutant has reduced growth and higher tolerance for drought, whereas overexpressing COST1 causes drought hypersensitivity and decreased autophagy [107]. Notably, *nbr1* mutant exhibits lower tolerance for drought and salt stress compared to *atg* mutants. However, it exhibits typical senescence when starved in the dark, proving that *AtNBR1* acts as an essential control point for the autophagic removal of toxic proteins during ER stress [105]. Elevated ATG gene expression under salt and drought stress were observed in various plants including apple, banana, barley, foxtail millet, peach, pepper, rice, and sweet orange [109].

Numerous abiotic stimuli have the ability to produce ROS, acting as a stress response signal molecule. In plant cells, mitochondria, plasma membrane, peroxisomes, and chloroplasts all generate ROS [104]. The accumulation of ROS is regulated by a balance between development and scavenging via an enzymatic or nonenzymatic mechanism in proteaphagy [99]. Abiotic stress and autophagy are linked via ROS. Tolerance to submersion and the activation of autophagy in plants, for instance, depend on plasma membrane-associated NADPH oxidase, a significant ROS generator [103]. In tomatoes, AOX can reduce the ROS surge brought by drought and as a result, reduced ROS levels encourage autophagy and tolerate drought. Autophagy supports the antioxidant system by destroying oxidative damaged organelles [100]. *Atg2* and *atg5* mutants show increased H_2_O_2_ buildup, which is consistent with the involvement of autophagy in oxidative stress. Moreover, *Arabidopsis* develops hypersensitivity to oxidative stress as a result of increased oxidative damage brought on by *AtATG18a* silencing [63]. Auto-phagosome induction and formation are deficient in *AtATG18* knockdown transgenic plants *RNAi-AtATG18a* [102]. Inactive catalase build up in clustered peroxisomes in autophagy-deficient seedlings, suggesting that pexophagy actively removes damaged peroxisomes. In response to various environmental challenges, ROS are important signaling molecules that encourage stomatal closure. In guard cells, the disruption of additional *ATGs*, such as *AtATG2*, *AtATG5*, *AtATG7*, *AtATG8*, *AtATG9*, *AtATG10*, or *AtATG12*, encourages the accumulation of peroxisomes. These mutants have decreased ROS scavenger catalase activity, which impairs stomatal opening in reaction to light and low CO_2_ levels [101].

Hypoxia in plants can lead to the induction of autophagy. ROS levels drastically increase in plants when they are submerged or overwatered, which induces autophagy. *Arabidopsis atg* mutants are more vulnerable to submersion because they produce more ROS and have higher PCD than WT plants. Other hypoxia and ethylene-responsive genes were expressed less in these mutants, whereas anaerobic respiration genes such as *ALCOHOL DEHYDROGENASE 1 (AtADH1)* and *PYRUVATE DECARBOXYLASE 1 (AtPDC1)* were expressed more [103]. Hypoxia cause oxidative stress by increase in ROS and in response, SnRK1 and AMPK activate autophagy. Plant resistance to submergence and autophagy activation needs ROS generation by Rboh (respiratory burst oxidase homolog) [60, 80]. In low-oxygen environments, nitric oxide (NO) promotes seed germination. It is possible that *SNITROSOGLUTATHIONE REDUCTASE1 (AtGSNOR1)*, which interacts with *AtATG8* to control seed germination in hypoxia, plays a variety of roles to hypoxic stress in plants [59]. In short, autophagy is vital cellular physiological process that works during reactions of plants to diverse abiotic stresses.

## 9. Conclusion and Future Prospects

Compared to mobile species such as animals and humans, plants are more susceptible to biotic and abiotic stresses. The global food security system is under threat from these stresses. For plants to cope with environmental challenges, numerous systems have emerged. Plant autophagy enhances a plant's ability to withstand stress and efficiently use nutrients, and this provides a potential means of helping plants to adapt climate change. Molecular mechanism of autophagy includes the identification of core autophagy machinery, which demonstrates its importance in biological responses and cellular homeostasis in plants. Recently, there has been a lot of interest in selective autophagy and functions to break down certain proteins and organelles under specific conditions. To comprehend how various environmental inputs are integrated and coordinated by autophagy in plant cells, regulatory network models of how autophagy in plants responds to stressors are needed in this era. Manipulation of autophagy and its genes can be a useful technique to improve nutrient usage efficiency and make the plants with better stress tolerance, leading to increased yield. Many concerns remain unsolved about the identity of autophagy receptors, how they engage cargo, and how competing catabolic processes are controlled. The molecular processes underlying selective autophagy pathways still require clarification. Involvement of autophagy in pathogen infection and immune response must be considered. It is still unclear how proteins govern autophagy, interact with other signaling pathways, and interact with phytohormones. The identification of the specific molecular pathways governing autophagy in a variety of plant species as well as the development of complex devices for the in vivo monitoring and control of autophagy in real time provide challenges in the study of plant autophagy. Furthermore, a major obstacle still exists in the translation of fundamental research findings into innovative agricultural practices to improve crop resilience and productivity.

## Figures and Tables

**Table 1:** Proteins involved in autophagy in yeast to *Arabidopsis thaliana*.

**Protein complexes**	**Proteins**	**Homologs functions in plants**	**References**
Autophagy induction and vesicle nucleation ATG1 complex	TOR	Protein kinase, negative activation of autophagy serine kinase phosphoprotein	[2, 4, 21–23]
	ATG1		
	ATG13		
	ATG 11		
	ATG101		

Autophagosome formation P13K complex	ATG6	VPS30	[1, 25, 26]
	ATG14	Protein kinase	
	VPS15	P13K	
	VPS34		

Ubiquitin-like conjugation (ATG12)	ATG5	For the conjugation of ATG12, it is a target	[27, 28, 32, 33]
	ATG7	For the conjugation of ATG12, it acts like E1 conjugating enzyme	
	ATG10	For the conjugation of ATG12, it acts like E2 conjugating enzyme	
	ATG12	Plays role in the ATG5 and ATG10 interaction. It is a modifier just like an ubiquitin	

Ubiquitin-like conjugation (ATG8)	ATG3	For the conjugation of ATG8, it acts like E2 conjugating enzyme	[27–30]
	ATG4	Cysteine protease	
	ATG7	For the conjugation of ATG8, it acts like E1 conjugating enzyme	
	ATG8	Plays role in the ATG3 interaction. It is a modifier just like an ubiquitin	

**Table 2:** Selective autophagy in plants.

**Sorts of selective autophagy**	**Selective cargoes**	**Receptors/adaptors of cargoes**	**Stress types**	**References**
Chlorophagy	Entire photo-damaged and RCBs, ATI-PS bodies	Not known yet	Darkness, irradiation such as UV-B, carbon starvation, strong light, and senescence	[6, 20, 38, 40, 56, 57]
Reticulophagy	Damaged ER	Not known yet	ER stress	[43, 44]
Mitophagy	Mitochondria	ATI1 and ATI2	Carbon starvation	[24, 33, 42]
Pexophagy (yeast)	Damaged/abnormal peroxisomes	NBR1, p62, ATG30, and ATG36	Normal growth conditions	[4, 36, 41]
Ribophagy	Ribosomes	Unknown	Nitrogen starvation	[36, 45, 46]
Proteaphagy	26S proteasome	RPN10 (UIMs)	Proteasome inhibitor treatment	[47–49]
Aggrephagy	Protein aggregates that are ubiquitylated and insoluble	ATG8 NBR1	High temperature stress	[37, 50, 51]
Xenophagy	CaMV and TuMV	NBR1	Virus infection	[52–55]

**Table 3:** Functions of autophagy in plants.

**Autophagy inductions**	**Functions of autophagy**	**Genes**	**Autophagy process**	**References**
Growth & development	Nutrients recycling	*AtATG5*, *AtATG7*, *AtATG8*	Nitrogen remobilization	[29, 31, 60, 61]
	Senescence	*AtATG2*, *AtATG5*, *AtATG7*, *AtATG8*, *AtATG9*, *AtATG18a*, *AtATG11*	SA pathway	[24, 62–64]
	Reproduction	*AtATG6/VPS30*, *AtVPS15*, *AtVPS34*, *NbATG2*, *OsATG7*	Pollen germination and seed production	[59, 65–70, 75]
	Lipid metabolism	*OsATG7*, *OsATG9*	Lipophagy	[65, 66]
	Root development	*PtATG8*	Tissue development	[71–74]

Biotic stress	Bio-trophic and necrotrophic pathogens	*NbATG6*, *NbVPS34*, *NbATG3*, *NbATG7*, *AtATG2*, *AtATG5*, *AtATG6*, *AtATG7*, *AtATG9*, *AtATG10*, *AtATG18a*	SA and JA pathways; HR‐PCD	[35, 47, 55, 76–83]
	Viruses	*AtATG5*, *AtATG7*, *AtATG8*, *AtNBR1*, *NbATG3*, *NbATG6*, *NbATG7*, *NbATG8*	ATG Protein's interaction with virulence factors	[54, 81, 84–87]

Abiotic stress	Nutrients starvation	*AtATG4*, *AtATG5*, *AtATG6*, *AtATG7*, *AtATG9*, *AtATG12, AtATG18a, MpATG18a, AtSnRK1 GmATG8c OsATG7*	Recycling of nitrogen and carbon	[20, 64, 88–93]
		*AtATG5, AtATG10, AtNBR1*	Recycling of micronutrients	[94–96]
	Heat stress	*AtATG5, AtATG7, AtATG8, AtNBR1, SlATG5, SlATG7, SlATG8, SlNBR1*	Autophagic clearance of insoluble ubiquitinated protein aggregates	[93–96]
	Cold stress	*TaATG8*	Lipid homeostasis	[97]
		*SlATG2, SlATG6, SlATG8, SlNBR1*	NBR1‐mediated selective Autophagy	[59, 98, 99]
	ROS stress (oxidation)	*AtATG18a AtATG2, AtATG5, AtATG7*, *AtATG8*, *AtATG9*, *AtATG10*, *AtATG12*	ROS clearance	[63, 99–104]
	Drought and salinity	*AtATG5*, *AtATG7*, *AtATG8*, *AtATG18a*, *OsATG10b*, *SlHsfA1a*, *AtCOST1*, *AtNBR1*, *MpATG18a*	ROS and oxidized products clearance	[3, 90, 100, 105–109]
	Hypoxia	*AtATG2*, *AtATG5*, *AtATG7*, *AtATG8*, *AtATG10*, *AtADH1*, *AtPDC1*, *AtGSNOR1*	ROS homeostasis	[59, 60, 80, 103]

## Data Availability

No data were used to support the findings of this study.
